# eHealth Literacy and Patient Portal Use and Attitudes: Cross-sectional Observational Study

**DOI:** 10.2196/40105

**Published:** 2023-01-27

**Authors:** Nikita Deshpande, Vineet M Arora, Hanna Vollbrecht, David O Meltzer, Valerie Press

**Affiliations:** 1 Pritzker School of Medicine University of Chicago Chicago, IL United States; 2 Section of General Internal Medicine Department of Medicine University of Chicago Chicago, IL United States; 3 Department of Medicine Brigham and Women’s Hospital Boston, MA United States; 4 Section of Hospital Medicine Department of Medicine University of Chicago Chicago, IL United States

**Keywords:** health literacy, patient portal, COVID-19, health technology, inpatients, digital health literacy, awareness, use, engagement, attitudes, hospitalized patients, access, accessibility, perception, health care delivery

## Abstract

**Background:**

Throughout the COVID-19 pandemic, patient portals have become more widely used tools of patient care delivery. However, not all individuals have equivalent access or ability to use patient portals.

**Objective:**

The aim of this study is to evaluate the relationships between eHealth literacy (eHL) and patient portal awareness, use, and attitudes among hospitalized patients.

**Methods:**

Inpatients completed patient portal surveys; eHL was assessed (eHealth Literacy Scale). Multivariable logistic regression analyses adjusted for age, self-reported race, gender, and educational attainment were completed with significance at *P*<.006 (Bonferroni correction).

**Results:**

Among 274 participants, most identified as Black (n=166, 61%) and female (n=140, 51%), mean age was 56.5 (SD 16.7) years, and 178 (65%) reported some college or higher educational attainment. One-quarter (n=79, 28%) had low eHL (mean 27, SD 9.5), which was associated with lower odds of portal access awareness (odds ratio 0.11, 95% CI 0.05-0.23; *P*<.001), having ever used portals (odds ratio 0.19, 95% CI 0.10-0.36; *P*<.001), less perceived usefulness of portals (odds ratio 0.20, 95% CI 0.10-0.38; *P*=.001), and lower likelihood of planning to use portals in the coming years (odds ratio 0.12, 95% CI 0.06-0.25; *P*<.001). As time through the COVID-19 pandemic passed, there was a trend toward increased perceived usefulness of patient portals (53% vs 62%, *P*=.08), but average eHL did not increase through time (*P*=.81).

**Conclusions:**

Low eHL was associated with less awareness, use, and perceived usefulness of portals. Perceived usefulness of portals likely increased through the COVID-19 pandemic, but patients’ eHL did not. Interventions tailored for patients with low eHL could ensure greater equity in health care delivery through the COVID-19 pandemic.

## Introduction

### Increasing Relevance of Patient Portals

Patient portals are increasingly important tools for providing patient care [1-6]. They are used to schedule appointments, view results, request medication refills, and communicate with health care professionals [1,6]. Recently, patient portals have become increasingly salient, playing a vital role in vaccine distribution [5], COVID test result notification [3], and maintenance of care [2,4] virtually through disruptions in service through the COVID-19 pandemic.

### Disparities With Portal Use and Access

As with all new technology, it is vital to assess how existing health and health care disparities are impacted by the growing use of these patient portals. Prior studies have found that some populations, such as individuals who identify as Hispanic or Black and individuals with lower educational attainment are less likely to access patient portals [7,8]. Furthermore, older patients have been found to be less likely to enroll in patient portal programs [1,7]. The digital divide describes disparities in individuals’ access to and capabilities to use technology and differences in outcomes when using technology. Key determinants of the divide have been shown to include age, educational attainment, and socioeconomic status [9-11].

### eHealth Literacy

eHealth literacy (eHL) characterizes patients’ ability to find, comprehend, and evaluate health information from electronic sources [12]. Patients with lower eHL have been found to use the internet less often and to be less likely to search for health information [13]. The eHealth Literacy Scale (eHEALS) has been validated in diverse patient populations and is a frequently used measure of eHL [14,15]. Similar to other tools, it has limitations, including lacking items measuring skills and comfort with navigating social media sites and peer support forums [16,17].

### Study Aim

Past study of patient portals has focused on the outpatient setting, but understanding portal and use and attitudes among admitted patients is also important and may capture a more impaired, high-risk patient population. To our knowledge, the relationship between eHL and patients’ engagement with portals has not been characterized among general medicine inpatient populations. This study aimed to characterize how age, self-reported race, and eHL were associated with portal awareness, use, and perceptions among adult inpatients at UChicago Medicine.

## Methods

### Study Design and Participant Population

Inclusion criteria were being 18 years or older, speaking English, and being admitted to a general medicine service. Patients who lacked decisional capacity due to altered mental status or some other conditions were excluded. The recruitment occurred during the daytime for eligible patients at any time during their hospitalization. Patients provided their consent to the trained research assistants who recruited patients and filled out demographic, eHL, and survey data on access to and use of technology, including patient portals.

### Ethical Considerations

This cross-sectional, observational survey was completed as a part of a larger quality of care study approved by the University of Chicago Biological Sciences Division institutional review board (#IRB16-0763).

### Data Collection and Analysis

According to previous literature, low eHL was considered <24 [13]. To evaluate technology use and access, participants were asked if they owned technological devices, if they had wireless internet at home, and how frequently they accessed the internet. To assess patient portal awareness and use, the participants were asked if they were aware of access to a patient portal and had used a patient portal in the past. To evaluate patient portal attitudes, participants were asked how confident they were in their ability to use a portal, how useful they believed a portal was, and how likely they would use a portal in the next year (Multimedia Appendix 1). The validated 8-item eHEALS tool assessed eHL [14]. The eHEALS tool asks patients about their ability and confidence in finding and discerning health information on the internet (Multimedia Appendix 2) [18]. Surveys were administered either in-person or over the phone. Cases with missing data were omitted.

Descriptive statistics included means, SDs, and proportions. Bivariate chi-squared analyses were conducted. Multivariable logistic regression analyses were performed to determine the differences in patient portal use and attitudes, adjusted for eHL (binary), age (binary, <65 vs ≥65), gender (binary), self-reported race (White, Black, and others), and education (high school diploma or less vs some college or more). A *P*<.006 defined statistical significance based on Bonferroni correction [19]. STATA (version 15.1; StataCorp LLC) was used for all analyses.

## Results

### Study Design and Participant Population

#### Study Enrollment

From January 11, 2020, to August 3, 2021, a total of 2795 patients were screened and 1957 (70%) were eligible. Of those eligible, 274 participants (14%) were enrolled and completed the survey. Demographic data of those who refused, were discharged before the approach, or were not available during the approach were not recorded. Overall, 93% (255/274) of surveys were administered over the phone.

#### Participant Characteristics

The mean age was 56.5 (SD 16.7) years. The majority of participants identified as Black (166/274, 61%) and female (140/274, 51%). Sixty-five percent (178/274) reported some college or higher educational attainment, 33% (90/274) reported at most a high school education, and 2% (6/274) did not know or declined to say (Table 1). The majority of participants did not know or declined to provide annual household income (190/274, 69%).

**Table 1:** Distributions and odds ratios for bivariable and multivariable logistic regressions predicting portal awareness, use, and attitudes.

			All participants (n=274), %		Low eHL^a^ (n=79), %		Adequate eHL (n=195), %		Bivariate *P* value		Multivariable odds ratios^b^		Multivariable *P* value
Age≥65 years			33		48		28		.002		N/A^c^		N/A
Females			51		46		53		.25		N/A		N/A
**Race**			N/A		N/A		N/A		.006		N/A		N/A
	White	26		13		31		N/A		N/A		N/A	
	Black	61		71		56		N/A		N/A		N/A	
	Others	14		16		13		N/A		N/A		N/A	
Some college or higher education			65		37		76		.001		N/A		N/A
Aware of portal access			77		43		90		<.001		0.11 (0.05, 0.23)		<.001
Portal usage ever			57		23		71		<.001		0.19 (0.10, 0.36)		<.001
Perceived portals as very useful			23		16		26		<.001		0.20 (0.10, 0.38)		.001
Likely to use portal in the next year			61		22		77		<.001		0.12 (0.06, 0.25)		<.001

^a^eHL: eHealth literacy.

^b^Multivariable odds ratios for 6 different regression models, each adjusting for age, sex, self-reported race, education, and eHL.

^c^N/A: not applicable.

### Data Collection and Analysis

#### Participant Technology Ownership, Use, and eHL

Most participants owned at least 1 technological device (260/274, 95%), had Wi-Fi access at home (219/274, 80%), and used the internet several times per day (192/274, 70%). Overall, 28% (79/274) of participants had low eHL (range 8-40; mean eHEALS score 27, SD 9.5).

#### Associated Factors of Portal Use and Attitudes

Low eHL (odds ratio [OR] 0.11, 95% CI 0.05-0.23; *P*<.001) and identifying as Black (OR 0.18, 95% CI 0.06-0.55; *P*=.002) were associated with lower odds of being aware of access to a portal (Table 1). Low eHL (OR 0.19, 95% CI 0.10-0.36; *P*<.001) was associated with lower odds of ever using a portal. Low eHL was associated with less perceived usefulness of patient portals (OR 0.20, 95% CI 0.10-0.38; *P*=.001). Older age (OR 0.31, 95% CI 1.73-5.95; *P*<.001) and low eHL (OR 0.12, 95% CI 0.06-0.25; *P*<.001) were associated with not planning to use portals in the coming year. The most common reasons why participants had not used portals in the past year included being unaware of their access (68/274, 25%), unable to set it up (27/274, 10%), and feeling it would not improve their health care experience (17/274, 6%).

### Changes in Portal Attitudes and eHL Through Time

Data were separated into quartiles based on survey administration date to evaluate trends over time. As time passed through the COVID-19 pandemic, there was a trend toward increased perceived usefulness of patient portals (53% [Q1] vs 62% [Q4]; *P*=.08), but average eHL did not increase through time (*P*=.81).

## Discussion

### Principal Findings

Low eHL was associated with less portal awareness and past use. It was additionally associated with more negative patient portal attitudes, including less perceived usefulness and less likelihood of planning to use a portal in the next year. Older age was also associated with lower odds of planning to use a portal in the future. While the COVID-19 pandemic resulted in trends toward increased perceived usefulness of portals through time, patients’ eHL did not increase through the pandemic, suggesting that the patients were not empowered to better use digital tools as the pandemic progressed.

These findings extend previous studies that the digital divide is shifting from a disparity in access to a disparity in digital capabilities (as measured by eHEALS) [20-22]. More than 90% (n =260) of patients in our sample had access to at least 1 technological device, but only two-thirds (n=195) had adequate eHL. Furthermore, this study extends the findings of correlation between eHL and patient portal use previously reported among outpatients and organ transplant recipients to a hospitalized, urban, predominantly Black general medicine population [23]. Studying eHL and portal attitudes among inpatients captures individuals during the unique stressor of hospitalization and patients who may not engage with outpatient medicine and may otherwise be missed. Future efforts to increase patient utilization of portals likely needs to shift from simply increasing access to the internet to other interventions such as increasing awareness of the usefulness of portals and interventions to assist patients with portal use, particularly among patients who are older and have low eHL [21].

### Addressing Low Portal Awareness and Use

Reported factors that prevented portal use were lack of awareness, difficulty with setup, and lack of belief in portal usefulness, rather than lack of technological access. Patient education can address some barriers to patient portal use. However, lower perceived usefulness and lower confidence in personal use are more complicated barriers, which may be addressed through modification of patient portal designs to be as intuitive and simple as possible [24]. Tools such as the Centers for Disease Control and Infection’s Clear Communication Index can be used to identify the effectiveness of web-based health information and has been used to assess quality in patient portals and improve their simplicity and clarity [25]. Furthermore, eHL screening and in-person introductions to portals may improve portal uptake [26,27].

### Study Limitations

Because of limited patient surveys administered before the onset of the COVID-19 pandemic, this study is underpowered to detect changes in portal uptake as a result of COVID-19. Other limitations of this study include relying on self-reported measures of technology access and past patient portal use, and that this study population did not include many individuals with technology access barriers. This single-site study represents patients of a large Midwestern, academic, urban medical center that may not be generalizable to suburban and rural patient populations in other regions or countries. Furthermore, generalizability to all inpatients may be limited as the sample was comprised primarily of adults hospitalized during the height of the COVID-19 pandemic. Finally, the use of eHEALS may fail to capture more dynamic, modern components of digital competency that newer scale measures such as the Digital Health Literacy Instrument, eHealth Literacy Questionnaire, and eHealth Literacy Assessment Toolkit [17,28].

### Conclusions

In conclusion, this study indicates that low eHL was strongly associated with decreased patient portal awareness, use, and more negative portal attitudes among adult hospitalized patients. As health care professionals increasingly rely on patient portals, eHL should be accounted for to ensure patients with lower literacy are not disproportionately disadvantaged. Future studies should aim to understand how patient portal design and provider communication surrounding patient portals can be optimized for patients with low eHL. Further investigation of what interventions increase individuals’ eHL may better equip patients to take advantage of growing health care technologies, although additional work on also empowering patients to do so is also needed.

## Appendix

Multimedia Appendix 1 Summary of patient portal survey items.

Multimedia Appendix 2 Summary of eHEALS scale items.

## Abbreviations

eHEALS: eHealth Literacy Scale
eHL: eHealth literacy
OR: odds ratio
