# Supplementary material for: eHealth Literacy and Patient Portal Use and Attitudes: Cross-sectional Observational Study
Source: JMIR Hum Factors. 2023 Jan 27;10:e40105. doi: 10.2196/40105 (PMC9919456; doi:10.2196/40105)
Supplement: Multimedia Appendix 1 [file humanfactors_v10i1e40105_app1.docx]

| Are you aware that you have access to an online patient portal (such as MyChart) through your health care organization? |
| --- |
| Which of the following tasks do you think would be easier with the use of an online patient portal? |
| Have you signed up for a patient portal ever? |
| Have you signed up for the patient portal through our health care system? |
| Have you used a patient portal to do anything ever? |
| Have you used a patient portal to do anything in the past year? |
| What stopped you from using a patient portal in the past year? |
| How often have you used a patient portal in the past year? |
| How useful do you think a patient portal would be in managing your health? |
| How useful has the patient portal been in managing your health? |
| How confident are you that you could access and use a patient portal? |
| In your opinion, has the patient portal made it easier or harder to manage your health? |
| How likely are you to continue using or start using a patient portal in the next year? |
