# Supplementary material for: eHealth Literacy and Patient Portal Use and Attitudes: Cross-sectional Observational Study
Source: JMIR Hum Factors. 2023 Jan 27;10:e40105. doi: 10.2196/40105 (PMC9919456; doi:10.2196/40105)
Supplement: Multimedia Appendix 2 [file humanfactors_v10i1e40105_app2.docx]

| I know how to find helpful health resources on the Internet |
| --- |
| I know how to use the internet to answer my health questions |
| I know what health resources are available on the Internet |
| I know where to find helpful health resources on the Internet |
| I know how to use the health information I find on the Internet to help me |
| I have the skills I need to evaluate the health resources I find on the internet |
| I can tell high quality from low quality health resources on the Internet |
| I feel confident in using information from the internet to make health decisions |
